# The Transmission Dynamics of a Compartmental Epidemic Model for COVID-19 with the Asymptomatic Population via Closed-Form Solutions

**DOI:** 10.3390/vaccines10122162

**Published:** 2022-12-16

**Authors:** Rehana Naz, Mariano Torrisi

**Affiliations:** 1 Department of Mathematics and Statistical Sciences, Lahore School of Economics, Lahore 53200, Pakistan; 2 Dipartimento di Matematica ed Informatica, UniversitÃ di Catania Viale A. Doria, 6, I-95125 Catania, Italy

**Keywords:** SAIQ model, closed-form solutions, COVID-19

## Abstract

Unlike previous viral diseases, COVID-19 has an “asymptomatic” group that has no symptoms but can still spread the disease to others at the same rate as symptomatic patients who are infected. In the literature, the mass action or standard incidence rates are considered for compartmental models with asymptomatic compartment for studying the transmission dynamics of COVID-19, but the quarantined adjusted incidence rate is not. To bridge this gap, we developed a Susceptible Asymptomatic Infectious Quarantined (SAIQ) model with a Quarantine-Adjusted (QA) incidence to investigate the emergence and containment of COVID-19. COVID-19 models are investigated using various methods, but only a few studies take into account closed-form solutions. The knowledge of closed-form solutions simplifies the construction of the various epidemic indicators that describe the epidemic phenomenon and makes the sensitivity analysis to variations in the data under consideration possible. The closed-form solutions of the systems of four nonlinear first-order ordinary differential equations (ODEs) are established. The Epidemic Peak (EP), Force of Infection (FOI) and Rate of Infection (ROI) are the important indicators for the control and prevention of disease. We examined these indicators using closed-form solutions and particular parameter values. Different disease control scenarios are thoroughly examined. The four scenarios to analyze COVID-19 propagation and containment are (i) lockdown, (ii) quarantine and other preventative measures, (iii) stabilizing the basic reproduction rate to a level where the pandemic can be contained and (iv) containing the epidemic through an appropriate combination of lockdown, quarantine and other preventative measures.

## 1. Introduction

The fundamental model of theoretical epidemiology, Susceptible–Infectious–Recovered (SIR) compartmental model of infectious disease, established by Kermack and Mckendrinck [1], is utilized by different researchers to understand the propagation and containment of COVID-19. It is worthy to mention here that in epidemiology, the population is split into compartments such as Susceptible (S), Infectious (I), Exposed (E), Recovered (R), Asymptomatic (A), Quarantined (Q), Vaccinated (V), Hospitalized (H), Diseased (D) and so on.

Several studies [2,3] on propagation and containment of COVID-19 reveal that a large number of infections are caused by asymptomatic infection. Robinson and Stilianakis [4] developed the SAIR model to capture asymptomatic infection. Ying and Xiaoqing [5], Kaushal et al. [6], Ansumali et al. [7] and Monteiro [8] studied the propagation of COVID-19 by using the SAIR model [4]. The quarantine is an effective measure to control the disease with a large number of asymptomatic groups [9]. The SIQS and SIQR models were studied by Hethcote et al. [10] to analyze the effects of quarantine for three different types of incidence functions. Naz and Al-Raeei [11] developed the SIQD model with a QA incidence to analyze the propagation of COVID-19. Bhadauria et al. [12] formulated the SIQ model to investigate the lockdown effect of COVID-19. The literature on the propagation and containment of COVID-19 is extensive. For a complete review of past research focusing on COVID-19 using the classical models SIR, SEIR and their extensions, the interested reader is directed to Massonis et al. [13] and references therein.

The epidemic models are investigated using a variety of methods, including global stability analysis, numerical methods and a data-driven approach. It is worthy to mention here that symmetry approaches have been successfully employed in the recent literature [14,15] to analyze models from biomathematics and other fields of applied mathematics [16,17,18,19,20]. Naz and Naeem [21] developed a new technique to construct first integrals and closed-form solutions of dynamical systems from epidemics. Several efficient computer packages [22,23,24,25,26] are developed for the computation of Lie symmetries for differential equations. In [27,28], the first integrals are utilized to construct the closed-form solutions of the SEI, SIRI and tuberculosis models. A separate strand of the literature focuses on the use of artificial intelligence (AI) and machine learning techniques in the management of COVID-19 disease. Keshavarzi et al. [29] provided an excellent survey of AI-based models for the development of COVID-19 vaccines and drugs. The information and datasets provided in this review can be utilized to accelerate the exploration of effective viral treatments. A web search engine misinformation notifier extension (SEMiNExt) proposed by Shams et al. [30] has enabled safer web-based searching on health-related issues by displaying misinformation notifications in real time.

COVID-19, unlike previous viral diseases, has an “asymptomatic” group that has no symptoms but can still spread the disease to others at the same rate as infected symptomatic patients. In this article, we focus only on four compartments: Susceptible S(t), Asymptomatic A(t), Infected I(t) and Quarantined Q(t). A SAIQ model with QA incidence is developed to investigate propagation and containment of COVID-19. The SAIQ model with a QA incidence has never been utilized in the literature to examine the propagation and containment of COVID-19 to our knowledge. COVID-19 models are investigated using various methods, but only a few studies focused on closed-form solutions. The closed-form solution facilitates the construction of the diverse epidemic indicators that describe the epidemic phenomenon and enables sensitivity analysis to variations in the data under consideration. We utilized the classical techniques of solving ODEs to establish the closed-form solutions of the systems of four nonlinear ODEs representing the SAIQ model. EP, FOI and ROI are vital epidemic indicators for the control and prevention of disease. We investigated these indicators using closed-form solutions. We provided four different scenarios to analyze COVID-19 19 propagation and containment: (i) lockdown, (ii) quarantine and other preventative measures, (iii) stabilizing the basic reproduction rate to a level where the pandemic can be contained and (iv) containing the epidemic through an appropriate combination of lockdown, quarantine and other preventative measures. It is worth noting that specific parameter values for any country could be used in the closed-form expressions for all the model variables, EP, FOI and ROI, derived here to analyze transmission dynamics and discuss control strategies.

The paper is organized in the following manner. The SAIQ model with QA incidence is established in Section 2. In Section 3, the dynamical system of four ODEs is reduced to a single second-order ODE in terms of only one variable *S*. The closed-form solutions for the variable *S* are obtained from the reduced second-order ODE, and then this is utilized to establish a closed-form solution for other variables of the model. In Section 4, the closed-form expressions of EP, FOI and ROI are derived by utilizing the closed-form solutions. In Section 5, specific values of parameters from the literature are used to construct graphs of closed-form expressions for EP, FOI, and ROI. Section 6 contains the final remarks.

## 2. The SAIQ Model with QA Incidence

A model to study the propagation and containment of COVID-19 is formulated which has four compartments: Susceptible S(t), Asymptomatic A(t), Infected I(t) and Quarantined Q(t), and the total population is N(t)=S(t)+A(t)+I(t)+Q(t). The rate of the efficient contact between susceptible and asymptomatic is the same as the rate of the efficient contact between susceptible and infected, and we take it as constant β>0. There is no obvious difference in viral shedding rates between asymptomatic and infected persons, according to the concept of viral shedding [7,31]. A QA incidence function [10,11] is utilized to analyze the transmission dynamics of COVID-19. The actively mixing population for the SAIQ model is N(t)−Q(t)=S(t)+A(t)+I(t). The QA incidence for the asymptomatic group is $\frac{\beta SA}{S+A+I}$ and for the infected group is $\frac{\beta SI}{S+A+I}$. The FOI for the asymptomatic group is $\frac{\beta A}{S+A+I}$ and the FOI for the infected group is $\frac{\beta I}{S+I+Q}$. Let δ>0 be the transfer rate between asymptomatic and infected, γ be the segregation rate of asymptomatic and infected individuals, and the quarantined individuals leave the quarantining compartment at a rate τ. The parameters of the model are summarized in Table 1.

The following system of differential equations describes the SAIQ model with infectious force in the asymptomatic and infected groups:

(1) $$\begin{array}{c} S^{\prime }=-\frac{\beta SA}{S+A+I}-\frac{\beta SI}{S+A+I}, \end{array}$$

(2) $$\begin{array}{c} A^{\prime }=\frac{\beta SA}{S+A+I}+\frac{\beta SI}{S+A+I}-\left(\delta +\gamma \right)A, \end{array}$$

(3) $$\begin{array}{c} I^{\prime }=\delta A-\gamma I, \end{array}$$

(4) $$\begin{array}{c} Q^{\prime }=\gamma \left(A+I\right)-\tau Q, \end{array}$$

with the initial conditions

(5) $$S\left(0\right)=S_{0},\;A\left(0\right)=A_{0},\;I\left(0\right)=I_{0},Q\left(0\right)=Q_{0}$$

here, S(t)+A(t)+I(t)+Q(t)=N(t), and prime represents the derivative of variable with respect to time. It is worthy to mention here that the population N(t) satisfies $N^{\prime }=-\tau Q$. Ansumali et al. [7] analyzed the SAIR model with standard incidence. Kaushal et al. [6] studied the SAIR model with migration effects. The quarantine is an effective measure to control disease with a large number of asymptomatic groups [9], and thus we replaced the removed/recovered *R* compartment by the quarantine compartment *Q*.

We utilize Driessche and Watmough’s [32] next-generation operator technique to obtain the basic reproduction number ρ. The disease-free equilibrium (DFE) is $S=S^{*},A^{*}=0,I^{*}=0,Q^{*}=0$ The diseased compartments are

(6) $$\left[\begin{array}{c} A^{\prime }\\ I^{\prime } \end{array}\right]=\left[\begin{array}{c} \frac{\beta SA}{S+A+I}+\frac{\beta SI}{S+A+I}\\ 0 \end{array}\right]-\left[\begin{array}{c} (\delta +\gamma )A\\ -\delta A+\gamma I \end{array}\right]$$

For the next-generation operator technique, the related *F* and *V* matrices of the model, computed at the DFE, are provided as

(7) $$F=\left[\begin{array}{c} \beta \\ 0 \end{array}\begin{array}{c} \beta \\ 0 \end{array}\right]$$

(8) $$V=\left[\begin{array}{c} \delta +\gamma \\ -\delta \end{array}\begin{array}{c} 0\\ \gamma \end{array}\right],$$

and

(9) $$FV^{-1}=\left[\begin{array}{c} \frac{\beta }{\gamma }\\ 0 \end{array}\begin{array}{c} \frac{\beta }{\gamma }\\ 0 \end{array}\right].$$

The eigen values of $FV^{-1}$ are

(10) $$\left[\begin{array}{c} 0\\ \frac{\beta }{\gamma } \end{array}\right]$$

The expression for the basic reproduction number ρ is

(11) $$\rho =\frac{\beta }{\gamma }.$$

ρ>1 indicates the beginning of the pandemic, ρ=1 implies EP and ρ<1 guarantees the disease’s end.

## 3. The Closed-Form Solution of SAIQ Model with QA Incidence

In this section, we derive the closed-form solution of the SAIQ model with QA incidence. First, the system of four ODEs is reduced to a second-order ODE in the single variable *S*. Then, we establish closed-form solution of the second-order ODE, and thus obtain a closed-form expression for the variable *S*. This results in simplification of the original model, and thus it becomes straightforward to derive solutions of other variables of the model. Equations (1) and () yield

(12) $$A^{\prime }+\left(\delta +\gamma \right)A=-S^{\prime },$$

and

(13) $$I=-\frac{\left(S+A\right)S^{\prime }+\beta SA}{S^{\prime }+\beta S}.$$

Equation (3) with the aid of Equations (12) and (13) yields

(14) $$\beta SS^{\prime \prime }+{S^{\prime }}^{2}\left(\gamma -2\beta \right)+\beta \left(\gamma -\beta \right)SS^{\prime }=0$$

where γ−β≠0 and γ−2β≠0, which implies ρ≠1 and $\rho \neq \frac{1}{2}$. We assume S≠0, then Equation (14) simplifies to

(15) $$\begin{array}{c} S^{\prime \prime }+\frac{\left(\gamma -2\beta \right)}{\beta }\frac{S^{\prime }}{S}+\left(\gamma -\beta \right)=0, \end{array}$$

and yields

(16) $$S={\left(\frac{c_{2}-c_{1}e^{-\left(\gamma -\beta \right)t}}{\beta }\right)}^{\frac{\beta }{\gamma -\beta }}.$$

To find the values of arbitrary constants, we employ the initial condition $S\left(0\right)=S_{0}$, which yields

(17) $$c_{2}=\beta S_{0}^{\frac{\gamma -\beta }{\beta }}+c_{1}.$$

Equations (16) with the aid of Equation (17) yields

(18) $$S\left(t\right)={\left[S_{0}^{\frac{\gamma -\beta }{\beta }}+\frac{c_{1}}{\beta }\left(1-e^{-\left(\gamma -\beta \right)t}\right)\right]}^{\frac{\beta }{\gamma -\beta }}.$$

It is important to note that Equation (1) has the following alternative form:

(19) $$A\left(t\right)+I\left(t\right)=-\frac{SS^{\prime }}{S^{\prime }+\beta S}.$$

We use *S* from Equation (18) in Equation (19) to obtain the following expression for A(t)+I(t):

(20) $$\begin{array}{c} A\left(t\right)+I\left(t\right)=\left(A_{0}+I_{0}\right){\left(\frac{S_{0}+A_{0}+I_{0}}{S_{0}}\right)}^{\frac{\beta }{\beta -\gamma }}\\ \times e^{(\beta -\gamma )t}{\left(1+\frac{A_{0}+I_{0}}{S_{0}}e^{(\beta -\gamma )t}\right)}^{-\frac{\beta }{\beta -\gamma }} \end{array}$$

where $A\left(0\right)=A_{0},\;I\left(0\right)=I_{0}$ and

(21) $$c_{1}=-\frac{A_{0}+I_{0}}{S_{0}+A_{0}+I_{0}}\beta S_{0}^{\frac{\gamma -\beta }{\beta }}.$$

Equations (3) and (20) yield

(22) $$\begin{array}{c} I^{\prime }+\left(\gamma +\delta \right)I=\delta \left(A_{0}+I_{0}\right){\left(\frac{S_{0}+A_{0}+I_{0}}{S_{0}}\right)}^{\frac{\beta }{\beta -\gamma }}\\ \times e^{(\beta -\gamma )t}{\left(1+\frac{A_{0}+I_{0}}{S_{0}}e^{(\beta -\gamma )t}\right)}^{-\frac{\beta }{\beta -\gamma }}. \end{array}$$

The solution of first-order liner ODE (22) is

(23) $$\begin{array}{c} I\left(t\right)=e^{-(\gamma +\delta )t}[I_{0}+\delta \left(A_{0}+I_{0}\right){\left(\frac{S_{0}+A_{0}+I_{0}}{S_{0}}\right)}^{\frac{\beta }{\beta -\gamma }}\\ \times \int_{0}^{t}e^{(\beta +\delta )t}{\left(1+\frac{A_{0}+I_{0}}{S_{0}}e^{(\beta -\gamma )t}\right)}^{-\frac{\beta }{\beta -\gamma }}dt], \end{array}$$

where $I\left(0\right)=I_{0}$.

Equations (4) and (20) yield

(24) $$\begin{array}{c} Q^{\prime }+\tau Q=\gamma \left(A_{0}+I_{0}\right){\left(\frac{S_{0}+A_{0}+I_{0}}{S_{0}}\right)}^{\frac{\beta }{\beta -\gamma }}\\ \times e^{(\beta -\gamma )t}{\left(1+\frac{A_{0}+I_{0}}{S_{0}}e^{(\beta -\gamma )t}\right)}^{-\frac{\beta }{\beta -\gamma }}. \end{array}$$

The solution of first-order liner ODE (24) is

(25) $$\begin{array}{c} Q\left(t\right)=e^{-\tau t}[Q_{0}+\gamma \left(A_{0}+I_{0}\right){\left(\frac{S_{0}+A_{0}+I_{0}}{S_{0}}\right)}^{\frac{\beta }{\beta -\gamma }}\\ \times \int_{0}^{t}e^{(\tau +\beta -\gamma )t}{\left(1+\frac{A_{0}+I_{0}}{S_{0}}e^{(\beta -\gamma )t}\right)}^{-\frac{\beta }{\beta -\gamma }}dt], \end{array}$$

where $Q\left(0\right)=Q_{0}$. The summary of the closed-form solution of the SAIQ model (1)–(4) is as follows:

(26) $$\begin{array}{c} S\left(t\right)=S_{0}{\left(\frac{S_{0}+A_{0}+I_{0}}{S_{0}}\right)}^{\frac{\beta }{\beta -\gamma }}{\left(1+\frac{A_{0}+I_{0}}{S_{0}}e^{(\beta -\gamma )t}\right)}^{-\frac{\beta }{\beta -\gamma }},\\ I\left(t\right)=e^{-(\gamma +\delta )t}[I_{0}+\delta \left(A_{0}+I_{0}\right){\left(\frac{S_{0}+A_{0}+I_{0}}{S_{0}}\right)}^{\frac{\beta }{\beta -\gamma }} \end{array}$$

(27) $$\begin{array}{c} \times \int_{0}^{t}e^{(\beta +\delta )t}{\left(1+\frac{A_{0}+I_{0}}{S_{0}}e^{(\beta -\gamma )t}\right)}^{-\frac{\beta }{\beta -\gamma }}dt],\\ A\left(t\right)=\left(A_{0}+I_{0}\right){\left(\frac{S_{0}+A_{0}+I_{0}}{S_{0}}\right)}^{\frac{\beta }{\beta -\gamma }}e^{(\beta -\gamma )t} \end{array}$$

(28) $$\begin{array}{c} \times {\left(1+\frac{A_{0}+I_{0}}{S_{0}}e^{(\beta -\gamma )t}\right)}^{-\frac{\beta }{\beta -\gamma }}-I\left(t\right),\\ Q\left(t\right)=e^{-\tau t}[Q_{0}+\gamma \left(A_{0}+I_{0}\right){\left(\frac{S_{0}+A_{0}+I_{0}}{S_{0}}\right)}^{\frac{\beta }{\beta -\gamma }} \end{array}$$

(29) $$\begin{array}{c} \times \int_{0}^{t}e^{(\tau +\beta -\gamma )t}{\left(1+\frac{A_{0}+I_{0}}{S_{0}}e^{(\beta -\gamma )t}\right)}^{-\frac{\beta }{\beta -\gamma }}dt], \end{array}$$

provided γ−β≠0 and γ−2β≠0, which implies ρ≠1 and $\rho \neq \frac{1}{2}$. The SAIQ model will reach the equilibrium value when β<γ, and thus ρ<1.

## 4. The Closed-Form Expressions of EP, FOI and ROI

In this section, we provide the closed-form expressions of EP, FOI and ROI.

### 4.1. The EP

The total number of infected individuals is P(t)=A(t)+I(t) and can be expressed in terms of ρ in the following form:

(30) $$\begin{array}{c} P\left(t\right)=\left(A_{0}+I_{0}\right){\left(\frac{S_{0}+A_{0}+I_{0}}{S_{0}}\right)}^{\frac{\rho }{\rho -1}}e^{\beta (\frac{\rho -1}{\rho })t}\\ \times {\left(1+\frac{A_{0}+I_{0}}{S_{0}}e^{\beta (\frac{\rho -1}{\rho })t}\right)}^{-\frac{\rho }{\rho -1}}. \end{array}$$

EP is determined from the total number of infected individuals P(t)=A(t)+I(t). The differentiation of P(t) given in (30) with respect to *t* yields

(31) $$\begin{array}{c} \frac{dP}{dt}=\frac{\beta }{\rho }\frac{S_{0}\left(\rho -1\right)-\left(A_{0}+I_{0}\right)e^{\beta (\frac{\rho -1}{\rho })t}}{\left(A_{0}+I_{0}\right)e^{\beta (\frac{\rho -1}{\rho })t}+S_{0}}\\ \times \left(A_{0}+I_{0}\right){\left(\frac{S_{0}+A_{0}+I_{0}}{S_{0}}\right)}^{\frac{\rho }{\rho -1}}{\left(1+\frac{A_{0}+I_{0}}{S_{0}}e^{\beta (\frac{\rho -1}{\rho })t}\right)}^{-\frac{\rho }{\rho -1}}e^{\beta (\frac{\rho -1}{\rho })t}. \end{array}$$

Setting $\frac{dP}{dt}=0$, we obtain

(32) $$t_{peak}=\ln{\left(\frac{S_{0}}{A_{0}+I_{0}}\left(\rho -1\right)\right)}^{\frac{\rho }{\beta (\rho -1)}},$$

and $t_{peak}>0$ as ρ>1 and $S_{0}>A_{0}+I_{0}$. The second-order derivative evaluated at $t_{peak}$ is

(33) $$\frac{d^{2}P}{dt^{2}}=-S_{0}\beta^{2}{\left(\frac{\rho -1}{\rho }\right)}^{3}{\left(\frac{S_{0}+A_{0}+I_{0}}{S_{0}\rho }\right)}^{\frac{\rho }{\rho -1}}.$$

Equation (33) indicates that $\frac{d^{2}P}{dt^{2}}<0$ as ρ>1. For this value of tpeak, the highest number of infected cases Ppeak is reported and is provided by

(34) $$P_{peak}=S_{0}\left(\rho -1\right){\left(\frac{S_{0}+A_{0}+I_{0}}{\rho S_{0}}\right)}^{\frac{\rho }{\rho -1}}.$$

### 4.2. FOI and ROI

The infectious force in the asymptomatic is $\Omega_{A}=\frac{\beta A}{S+A+I}$, and the infectious force in the infected group is $\Omega_{I}=\frac{\beta I}{S+A+I}$. The total infectious force is $\Omega =\Omega_{A}+\Omega_{I}$ and is given by

(35) $$\Omega =\frac{\beta (A+I)}{S+A+I}=\frac{\beta }{1+\frac{S}{P}}.$$

The closed-from expression for the susceptible S(t) (26) can be expressed in terms of ρ as follows:

(36) $$S\left(t\right)=S_{0}{\left(\frac{S_{0}+A_{0}+I_{0}}{S_{0}}\right)}^{\frac{\rho }{\rho -1}}{\left(1+\frac{A_{0}+I_{0}}{S_{0}}e^{\beta (\frac{\rho -1}{\rho })t}\right)}^{-\frac{\rho }{\rho -1}}.$$

Equation (35) has the following form with the help of equations (30) and (36):

(37) $$\Omega \left(t\right)=\beta \left(\frac{A_{0}+I_{0}}{S_{0}}\right)\frac{e^{\beta (\frac{\rho -1}{\rho })t}}{1+\frac{A_{0}+I_{0}}{S_{0}}e^{\beta (\frac{\rho -1}{\rho })t}}.$$

The ROI is defined as Φ=Ω(t)S(t) and is given by

(38) $$\begin{array}{c} \Phi \left(t\right)=\beta \left(A_{0}+I_{0}\right){\left(\frac{S_{0}+A_{0}+I_{0}}{S_{0}}\right)}^{\frac{\rho }{\rho -1}}\\ \times e^{\beta (\frac{\rho -1}{\rho })t}{\left(1+\frac{A_{0}+I_{0}}{S_{0}}e^{\beta (\frac{\rho -1}{\rho })t}\right)}^{-\frac{\rho }{\rho -1}-1}. \end{array}$$

## 5. Analysis of EP, FOI and ROI to Analyze Propagation and Containment of COVID-19

In this section, we explore the results obtained in Section 4 using the specific values of parameters from the existing literature [5,6,7,8] and [33,34]. The total number of infected individuals P(t)=A(t)+I(t), the time span to reach at the epidemic peak $t_{peak}$, the epidemic peak $P_{peak}$, and the FOI Ω(t) and ROI Φ(t) are important indicators to study the propagation and containment of epidemics. It is important to mention here that the closed-form expressions of the total number of infected individuals (30), the time span to reach at the epidemic peak (32), the EP (34), FOI (37) and ROI (38) can be applied to any country’s real data to analyze the propagation and containment of epidemics. This can be achieved by inserting the parameters values and initial conditions for a specific country in these expressions.

We provide different scenarios to manage the epidemic through lockdown, quarantine and other preventative measures.

### 5.1. Scenario I: To Contain the Epidemic through Lockdown

**Figure 1:**
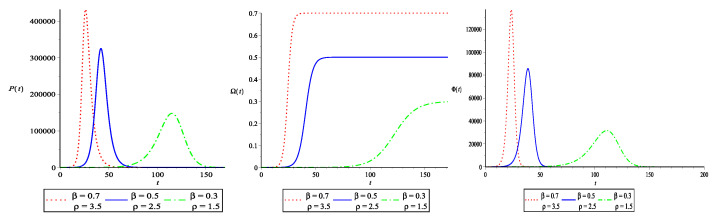
The effect of lockdown on EP, FOI and ROI for $N=10^{6}$ and 0≤t≤200. The value of γ=0.2 is fixed and, to lower the basic reproduction number, the value of β is reduced. We used $S\left(0\right)=10^{6}-5$, A(0)=2, I(0)=3 and Q(0)=0. The parameters are measured in (day${}^{-1})$. The time *t* is taken on the horizontal axis and is measured in days.

In the first scenario, a lockdown policy is implemented to contain the virus. The segregation rate for asymptomatic and infected people γ is set at 0.2. By lowering the rate of efficient contact β between susceptible and asymptomatic/infected, the basic reproduction number ρ is reduced. Figure 1 illustrates this scenario graphically. With a reduction in β, the asymptomatic and infected people curve P(t) takes longer to reach its maximum value. The EP gets smaller, and the time it takes to reach there gets longer. With a reduction in β, the FOI and the ROI both slow down. This suggests that lockdown is effective in minimizing FOI and ROI for a limited period of time but at the cost of social and economic consequences. In the long run, this is not a viable method for disease control.

### 5.2. Scenario II: To Contain the Epidemic through Quarantine and Other Preventative Measures

**Figure 2:**
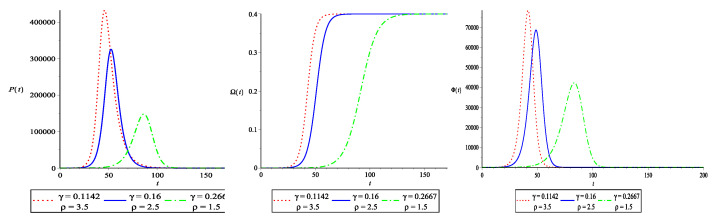
The effect of quarantine and other preventative measures on EP, FOI and ROI for $N=10^{6}$ and 0≤t≤200. The value of β=0.4 is fixed and, to lower ρ, the value of γ is increased. We used $S\left(0\right)=10^{6}-5$, A(0)=2, I(0)=3 and Q(0)=0. The parameters are measured in (day${}^{-1})$. The time *t* is taken on the horizontal axis and is measured in days.

In the second scenario, the virus is contained through quarantine and other preventative measures. The effective contact rate between susceptible and asymptomatic/infected β is set at 0.4. The segregation rate of asymptomatic and infected groups γ is increased to lower the basic reproduction number. Figure 2 illustrates this scenario visually. With an increase in γ, the asymptomatic and infected individuals curve P(t) takes longer to reach its maximum value. The EP gets smaller, and the time it takes to reach there gets longer. The strength of the FOI remains the same as γ rises (quarantine and preventive), but the time it takes to get at a certain point rises. With an increase in γ, the transfer rate of the susceptible to asymptomatic/infected group is lowered. This indicates that the community is adhering to the quarantine, social distancing and preventive measures in place. This is an excellent technique for preventing disease transmission.

### 5.3. Scenario III: To Stabilize the Basic Reproduction Rate down to a Level where the Pandemic Can Be Contained

**Figure 3:**
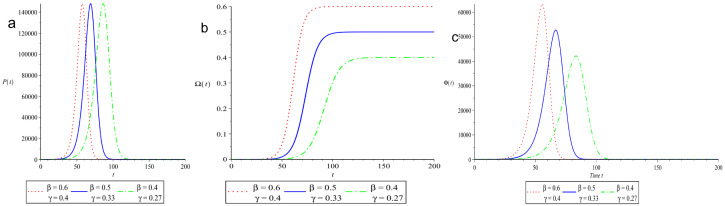
The behavior of EP, FOI and ROI for varying β and γ (with a fixed reproduction number ρ=1.5). We used $S\left(0\right)=10^{6}-5$, A(0)=2, I(0)=3, Q(0)=0, $N=10^{6}$ and 0≤t≤200. The parameters are measured in (day${}^{-1})$. The time *t* is taken on the horizontal axis and is measured in days.

Another effective scenario for controlling disease transmission is to fix ρ close to the level where transmissibility patterns are still moderate. A reduction in β leads to a decrease in γ for a specific value of ρ=1.5. Figure 3a–c shows the impact of changing β and γ with a given reproduction number on EP, FOI and ROI. The EP remains the same since the basic reproduction number is fixed, but the time it takes to reach it increases as β and γ drop, as seen in Figure 3a. Figure 3b,c shows how decreasing β and γ slows down the FOI and ROI. Instead of enforcing a strict lockdown to immediately flatten the curve, it is vital to decelerate disease propagation to prevent a new epidemic wave. This is a suitable approach to utilize the health care facilities for asymptomatic or symptomatic individuals P* for the time period $t_{*}$.

### 5.4. Scenario IV: To Contain the Epidemic through Appropriate Combination of Lockdown, Quarantine and Other Preventative Measures

**Figure 4:**
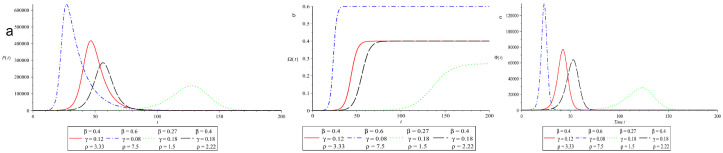
Effect of change in varying β and γ (in an appropriate combination to lower reproduction number) on epidemic peak, FOI and ROI for $N=10^{6}$ and 0≤t≤200. We used $S\left(0\right)=10^{6}-5$, A(0)=2, I(0)=3 and Q(0)=0. The parameters are measured in (day${}^{-1})$. The time *t* is taken on the horizontal axis and is measured in days.

Another scenario for containing the outbreak is to use a mix of lockdown, quarantine and other preventive measures. This may be accomplished by reducing the value of ρ by adjusting the effective contact rate between susceptible and asymptomatic/infected β and the segregation rate of asymptomatic/infected individuals γ in the right proportions. We started with bench mark values ρ=3.33, β=0.4 and γ=0.12, as indicated in the red solid line in Figure 4. The EP is on day 47 with 417,530 infected individuals. The parameters were then adjusted by a fixed proportion ϕ=1.5. The EP, FOI and ROI are then assessed for new parameter values and compared with those at benchmark parameter values.

We start by looking at consequences of entirely ignoring lockdown, quarantine and other preventive measures. This indicates there is a higher β in the effective contact rate between susceptible and asymptomatic/infected. Asymptomatic/infected people are isolated at a lower rate γ. When we increase β to ϕβ and reduce γ to γ/ϕ. This is depicted as a blue dashed line in Figure 4a–c. The value of ρ is 7.5, the EP is on day 27 with 635,663 number of asymtomatic/infected individuals P(t). The FOI and ROI rise at a faster pace, as seen in Figure 4b,c in the blue dashed line. This implies that a lockdown, quarantine and other preventative measures are necessary to contain the outbreak.

Next, we examine the impact of lockdown, quarantine and other preventive measures. This indicates that the effective contact rate β between susceptible and asymptomatic/infected should be reduced. Asymptomatic/infected people should isolate at a higher rate γ. As a result, we lower β to β/ϕ and raise γ to ϕγ. This is provided in Figure 4a–c in the green dotted line. With a value of ρ=1.5, the EP occurs on day 128, with a total of 148,149 asymtomatic/infected individuals P(t). The FOI and ROI rise at a slower pace for a longer duration, as seen in Figure 4b,c in the green dotted line. The pandemic peak shrinks and the time it takes to achieve there increases. The slow disease propagation is useful because the government will have ample time to strengthen the country’s healthcare infrastructure in order to meet the needs of a significant number of patients expected in the near future. This is the optimal scenario for reducing EP while extending the time it takes to reach epidemic peak.

Finally, we investigate the dynamics of epidemic transmission by increasing quarantine and other preventative measures without enforcing lockdown. This indicates that the asymptomatic/infected isolation rate γ should be increased while keeping the effective contact rate β between susceptible and asymptomatic/infected at a fixed level. This is established by increasing γ to ϕγ while keeping β constant. This is graphically represented in Figure 4a–c in the black long-dash line. With a value of ρ=2.22, the EP appears on day 56, with a total of 285833 asymtomatic/infected individuals. The strength of the FOI remains the same, but the time span to get at a specific point increases, as seen in Figure 4b in the black long-dash line. The transfer rate of susceptible individuals to asymptomatic/infected class is lowered, as seen in Figure 4c in the black long-dash line. This is another optimal scenario for reducing EP while extending the time it takes to reach EP without enforcing lockdown.

## 6. Conclusions

We established a SAIQ model with a QA incidence to analyze the propagation and containment of COVID-19. The closed-form solutions of the systems of four nonlinear first-order ODEs were established after reducing the system to a single ordinary differential equation in terms of the variable *S*. We provided the expressions for the important indicators of disease in the closed form. The propagation pattern and containment strategies of COVID19 are discussed using the numerical values of parameters from the literature in closed-form expressions of total number of infections P(t)=A(t)+I(t), FOI Ω(t) and ROI Φ(t).

In the first scenario, a lockdown policy is implemented to prevent the spread of a virus. We concluded that lockdown is a good strategy for a short period in lowering FOI and ROI but at the expense of social and economic losses. In the long run, this is not a viable method for disease control. In the second scenario, the virus is contained through quarantine and other preventative measures. The EP gets smaller, and the time it takes to reach there gets longer. The strength of the FOI remains the same as the community adheres to the quarantine, social distancing and other preventive measures, but the time span to get at a specific point increases. The transfer rate of the susceptible to asymptomatic/infected class is lowered. This is an excellent technique for preventing disease transmission. The third scenario for controlling disease transmission is to fix the value of ρ close to the value where disease propagation is moderate. The EP remains the same since the basic reproduction number is fixed, but the time it takes to reach it increases as β and γ drop. The decreasing β and γ slows down the FOI and ROI. Instead of enforcing a strict lockdown to immediately flatten the curve, it is vital to slow down disease propagation to prevent a new epidemic wave. This is a suitable approach to utilize the healthcare facilities for asymptomatic or symptomatic individuals P* for the time period $t_{*}$. Another scenario for containing the outbreak is to use a mix of lockdown, quarantine and preventive measures. This may be accomplished by reducing the value of the ρ by adjusting the effective contact rate between susceptible and asymptomatic/infected β and the segregation rate of asymptomatic/infected individuals γ in the right proportions.

In the majority of countries, government-implemented lockdown measures have significant social and economic implications. We discovered that quarantine and preventative measures are more successful in slowing disease propagation than harsh lockdown. Slow disease propagation can aid a country’s healthcare infrastructure. Implementing a partial shutdown with sufficient quarantine and preventative measures is the best way to cope with the pandemic.

It is worth mentioning that by including a compartment of vaccinated individuals to the model, the model could be modified to take vaccine effectiveness into consideration and forecast the spread of infection to demonstrate how vaccination could control an epidemic. This is a significant topic of epidemic control research and will be explored in a future article.

## Figures and Tables

**Table 1:** Model parameters.

Parameter	Description
β	efficient contact between susceptible and asymptomatic/infected
δ	transfer rate between asymptomatic and infected
γ	segregation rate of asymptomatic and infected individuals
τ	rate at which the quarantined individuals leave the quarantining compartment

## Data Availability

Data sharing not applicable to this article as no datasets were generated or analyzed during the current study.

## References

[1] Kermack W.O., McKendrick A.G. (1927). A contribution to the mathematical theory of epidemics. R. Soc. Lond. A.

[2] Oran D.P., Topol E.J. (2020). Prevalence of asymptomatic SARS-CoV-2 infection: A narrative review. Ann. Intern. Med..

[3] Day M. (2020). COVID-19: Identifying and isolating asymptomatic people helped eliminate virus in Italian village. BMJ Br. Med. J. Online.

[4] Robinson M., Stilianakis N.I. (2013). A model for the emergence of drug resistance in the presence of asymptomatic infections. Math. Biosci..

[5] Ying L., Xiaoqing T. (2021). COVID-19: Is it safe now? Study of asymptomatic infection spread and quantity risk based on SAIR model. Chaos Solitons Fractals X.

[6] Kaushal S., Rajput A.S., Bhattacharya S., Vidyasagar M., Kumar A., Prakash M.K., Ansumali S. (2020). Estimating the herd immunity threshold by accounting for the hidden asymptomatics using a COVID-19 specific model. PLoS ONE.

[7] Ansumali S., Kaushal S., Kumar A., Prakash M.K., Vidyasagar M. (2020). Modelling a pandemic with asymptomatic patients, impact of lockdown and herd immunity, with applications to SARS-CoV-2. Annu. Rev. Control.

[8] Monteiro L.H.A. (2020). An epidemiological model for SARS-CoV-2. Ecol. Complex..

[9] Fraser C., Riley S., Anderson R.M., Ferguson N.M. (2004). Factors that make an infectious disease outbreak controllable. Proc. Natl. Acad. Sci. USA.

[10] Hethcote H., Zhien M., Shengbing L. (2002). Effects of quarantine in six endemic models for infectious diseases. Math. Biosci..

[11] Naz R., Al-Raeei M. (2021). Analysis of transmission dynamics of COVID-19 via exact solutions of a susceptible-infectious-quarantined-diseased model with a QA incidence. Math. Methods Appl. Sci..

[12] Bhadauria A.S., Pathak R., Chaudhary M. (2021). A SIQ mathematical model on COVID-19 investigating the lockdown effect. Infect. Dis. Model..

[13] Massonis G., Banga J.R., Villaverde A.F. (2020). Structural identifiability and observability of compartmental models of the COVID-19 pandemic. Annu. Rev. Control.

[14] Freire I.L., Torrisi M. (2013). Symmetry methods in mathematical modeling of Aedes aegypti dispersal dynamics. Nonlinear Anal. Real World Appl..

[15] Naz R., Torrisi M. (2022). Symmetry methods for a hyperbolic model for a class of populations. Appl. Math. Comput..

[16] Torrisi M. (1988). Similarity solutions and wave prpagation in a reactive poly tropic gas. J. Eng. Math..

[17] Freire I.L., Da Silva P.L., Torrisi M. (2013). Lie and Nöther symmetries for a class of fourth order Emden Fowler equations. J. Phys. A Math. Theor..

[18] Muatjetjeja B., Khalique C.M. (2014). Lie group classification for a generalised coupled Lane-Emden system in dimension one. East Asian J. Appl. Math..

[19] Muatjetjeja B., Khalique C.M. (2014). A variational formulation approach to a generalized coupled inhomogeneous Emdenâ€“Fowler system. Appl. Anal..

[20] Muatjetjeja B. (2017). Coupled Laneâ€“Emdenâ€“Kleinâ€“Gordonâ€“Fock system with central symmetry: Symmetries and conservation laws. J. Differ. Equ..

[21] Naz R., Naeem I. (2018). The artificial Hamiltonian, first integrals, and closed-form solutions of dynamical systems for epidemics. Z. fur Naturforschung.

[22] Champagne B., Hereman W., Winternitz P. (1991). The computer calculation of Lie point symmetries of large systems of differential equations. Comput. Phys. Commun..

[23] Hereman W. (1993). SYMMGRP. MAX and other symbolic programs for lie symmetry analysis of partial differential equation. Lect. Appl. Math..

[24] Rocha Filho T.M., Figueiredo A. (2011). [SADE] a Maple package for the symmetry analysis of differential equations. Comput. Phys. Commun..

[25] Hereman W. (1996). Symbolic software for Lie symmetry analysis. CRC Handbook of Lie Group Analysis of Differential Equations.

[26] Hereman W. (1997). Review of symbolic software for Lie symmetry analysis. Math. Comput. Model..

[27] Naz R., Torrisi M. (2022). The first integrals and closed-form solutions of a Susceptible-Exposed-Infectious epidemic model. Math. Methods Appl. Sci..

[28] Naz R., Mahomed K.S., Naeem I. (2016). First integrals and exact solutions of the SIRI and tuberculosis models. Math. Methods Appl. Sci..

[29] Keshavarzi Arshadi A., Webb J., Salem M., Cruz E., Calad-Thomson S., Ghadirian N., Yuan J.S. (2020). Artificial intelligence for COVID-19 drug discovery and vaccine development. Front. Artif. Intell..

[30] Shams A.B., Hoque Apu E., Rahman A., Sarker Raihan M.M., Siddika N., Preo R.B., Kabir R. (2021). Web search engine misinformation notifier extension (SEMiNExt): A machine learning based approach during COVID-19 Pandemic. Healthcare.

[31] He X., Lau E.H., Wu P., Deng X., Wang J., Hao X., Lau Y.C., Wong J.Y., Guan Y., Tan X. (2020). Temporal dynamics in viral shedding and transmissibility of COVID-19. Nat. Med..

[32] Van den Driessche P., Watmough J. (2002). Reproduction numbers and sub-threshold endemic equilibria for compartmental models of disease transmission. Math. Biosci..

[33] Locatelli I., Trächsel B., Rousson V. (2021). Estimating the basic reproduction number for COVID-19 in Western Europe. PLoS ONE.

[34] Chisholm R.H., Campbell P.T., Wu Y., Tong S.Y., McVernon J., Geard N. (2018). Implications of asymptomatic carriers for infectious disease transmission and control. R. Soc. Open Sci..

